# Supplementary figures and images for: Artificial trans-kingdom RNAi of FolRDR1 is a potential strategy to control tomato wilt disease
Source: PLoS Pathog. 2023 Jun 20;19(6):e1011463. doi: 10.1371/journal.ppat.1011463 (PMC10313012; doi:10.1371/journal.ppat.1011463)

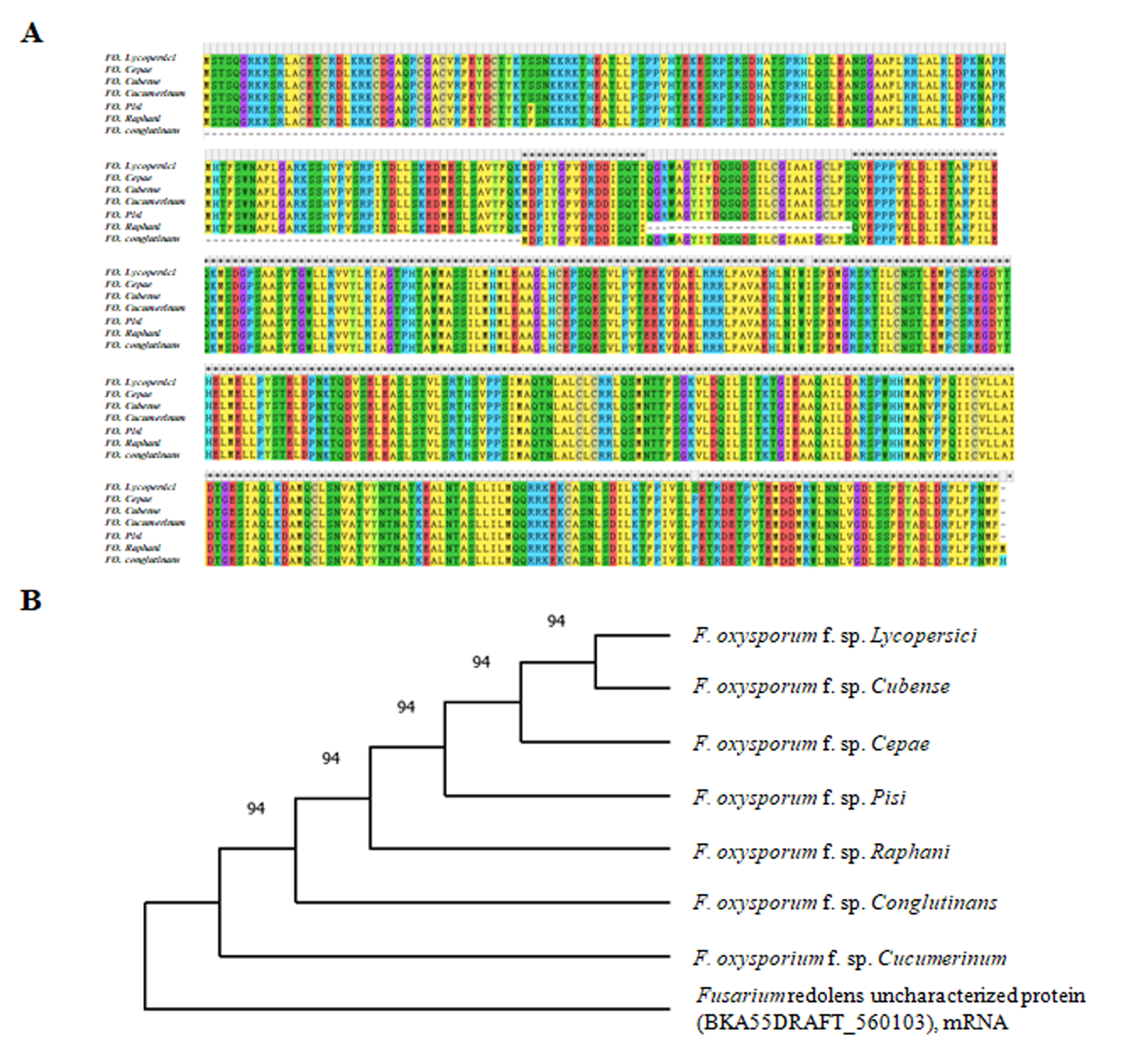

Supplement: S1 Fig — A Alignment of amino acid sequence of RDR1 using Pairwise Align Protein. All amino acid sequence of RDR1 were from https://www.ncbi.nlm.nih.gov. B The phylogenetic tree was constructed using MEGA (Molecular Evolutionary Genetics Analysis). (TIF) [file ppat.1011463.s001.tif]

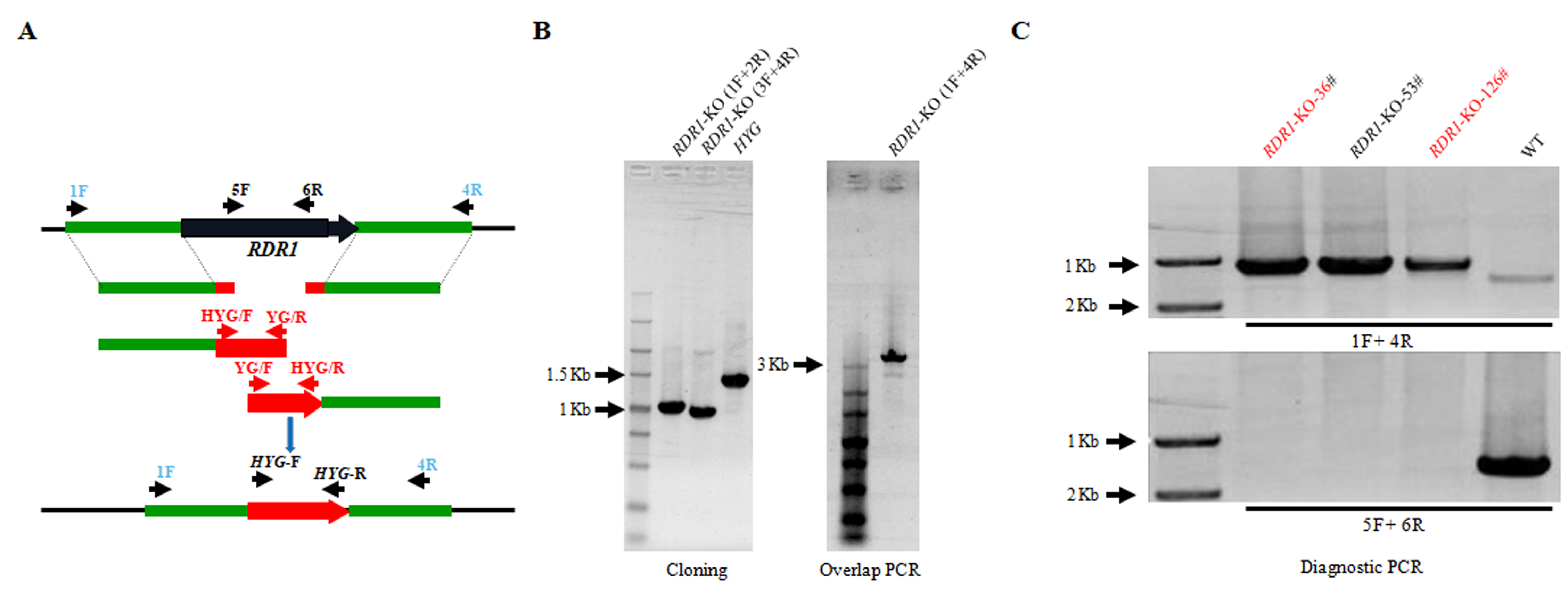

Supplement: S2 Fig — A Concise schematic diagram of homologous recombination. B PCR fragments used for homologous recombination. C Diagnostic PCR was used to identify positive clones. (TIF) [file ppat.1011463.s002.tif]

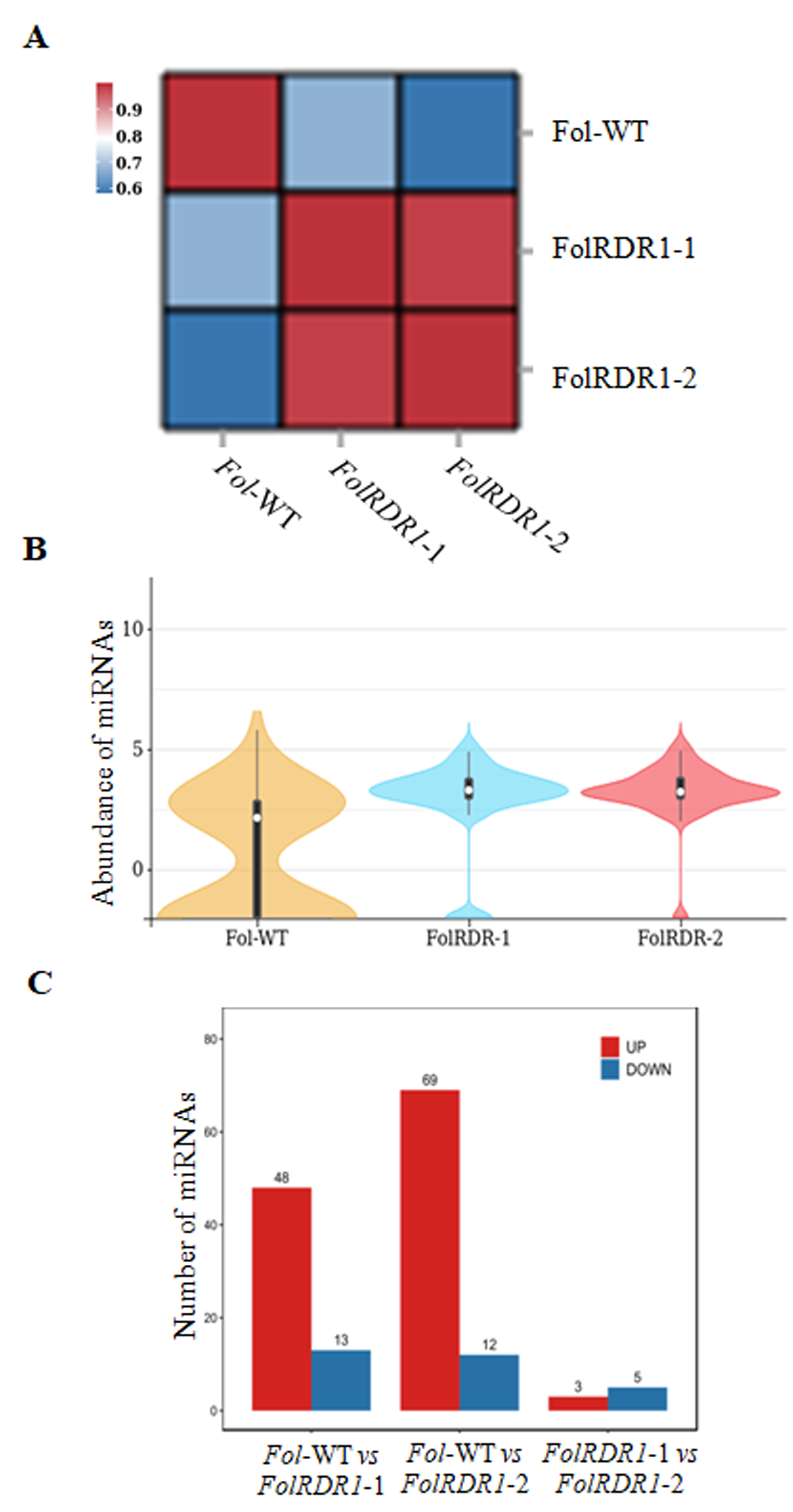

Supplement: S3 Fig — In the library, KO-strains FolRDR1-KO-36 was named as FolRDR1-1, FolRDR1-KO-126 was named as FolRDR1-2, and wild type strain was named as Fol-WT. A Correlation heat map analysis. B, C The abundances of miRNAs declined in both FolRDR1-KO strains. (TIF) [file ppat.1011463.s003.tif]

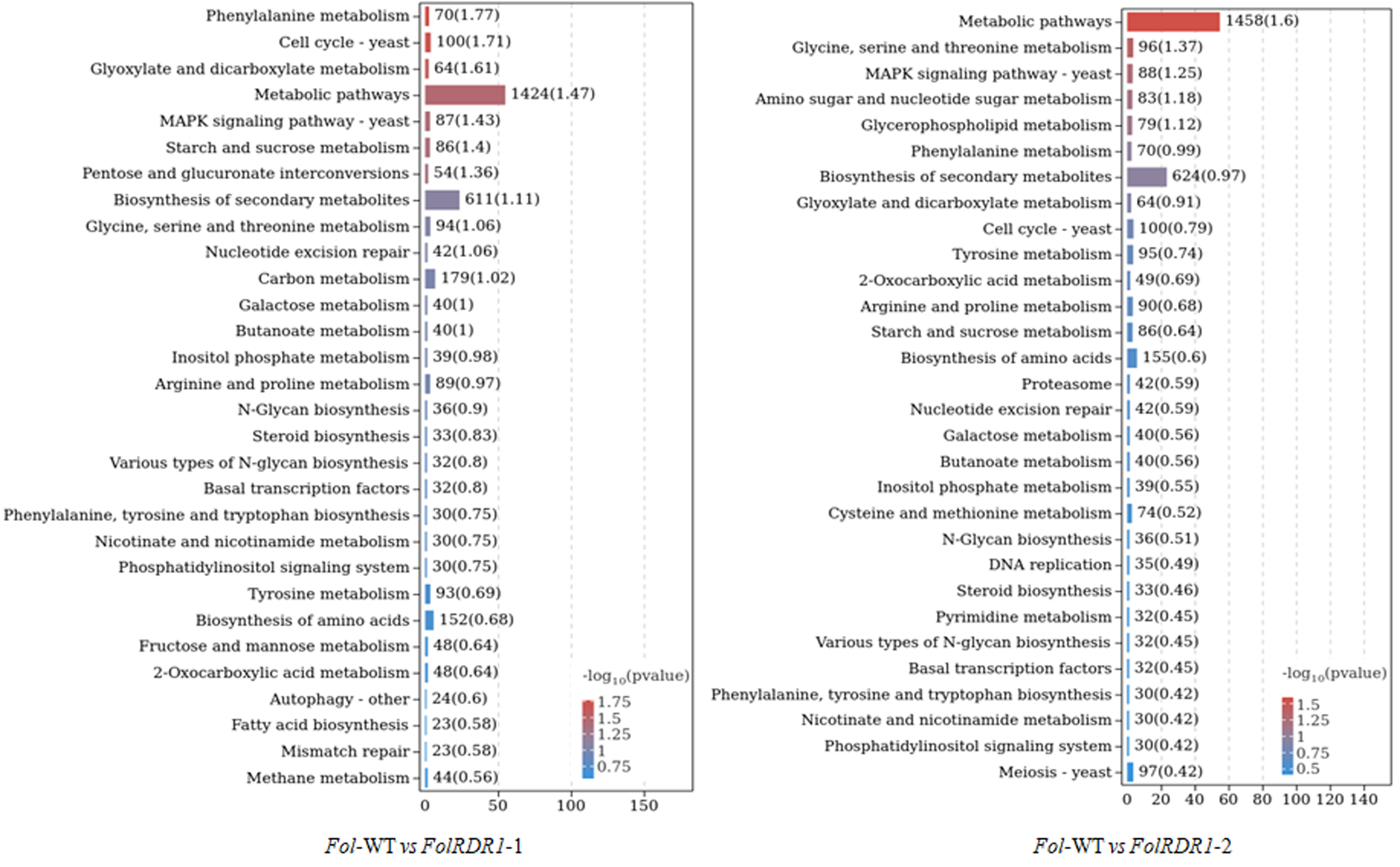

Supplement: S4 Fig — Knockouting of FolRDR1 mainly affected the metabolic pathway in both KO strains. (TIF) [file ppat.1011463.s004.tif]

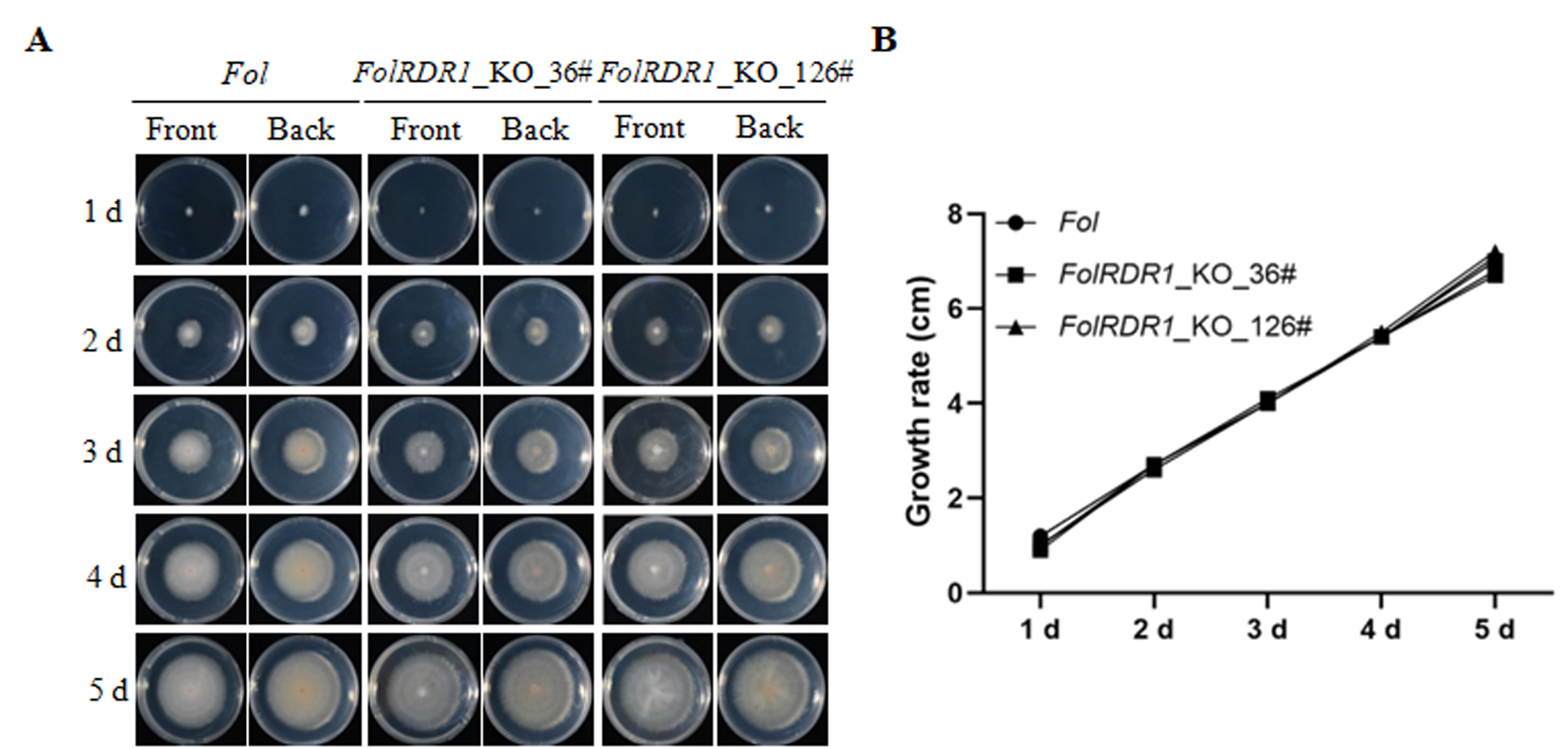

Supplement: S5 Fig — A All strains were culture on PDA plate, and photographed at different time points. B The growth curve was generated based on the colony diameter. Front, images were taken from the front of plate. Back, images were taken from the back of plate. Three biological replicates were used in each experiment. (TIF) [file ppat.1011463.s005.tif]

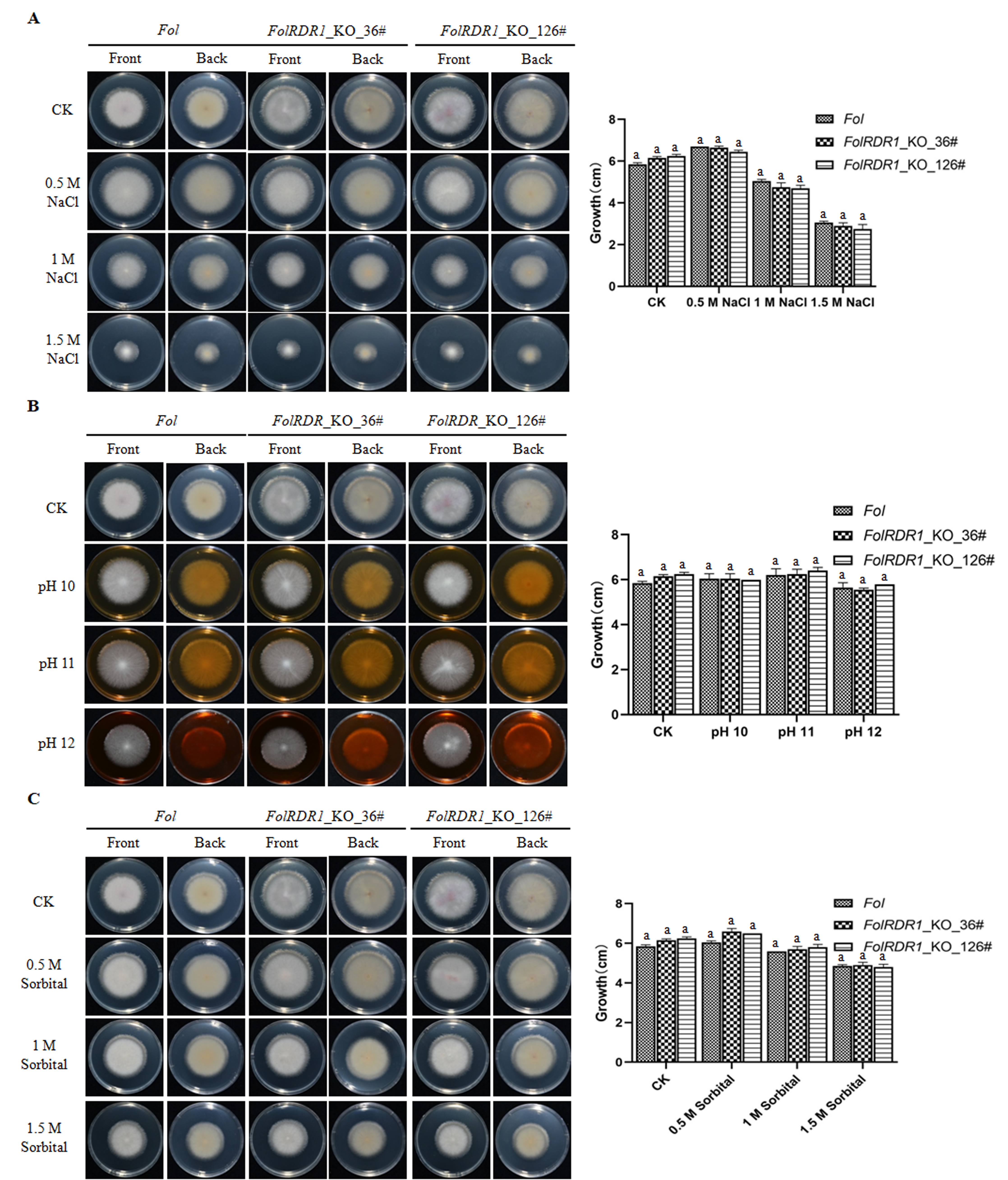

Supplement: S6 Fig — A All strains were culture on PDA plate with different concentration of NaCl (Left). The growth of colony was scaled at different time points, and the growth curve was generated (Right). B All strains were culture on PDA plate with different pH (Left). The growth of colony was scaled at different time points, and the growth curve was generated (Right). C All strains were culture on PDA plate with different concentration of sorbital (Left). The growth of colony was scaled at different time points, and the growth curve was generated (Right). Front, images were taken from the front of plate. Back, images were taken from the back of plate. Three biological replicates were used in each experiment. a presents no significant differences (p>0.05). (TIF) [file ppat.1011463.s006.tif]

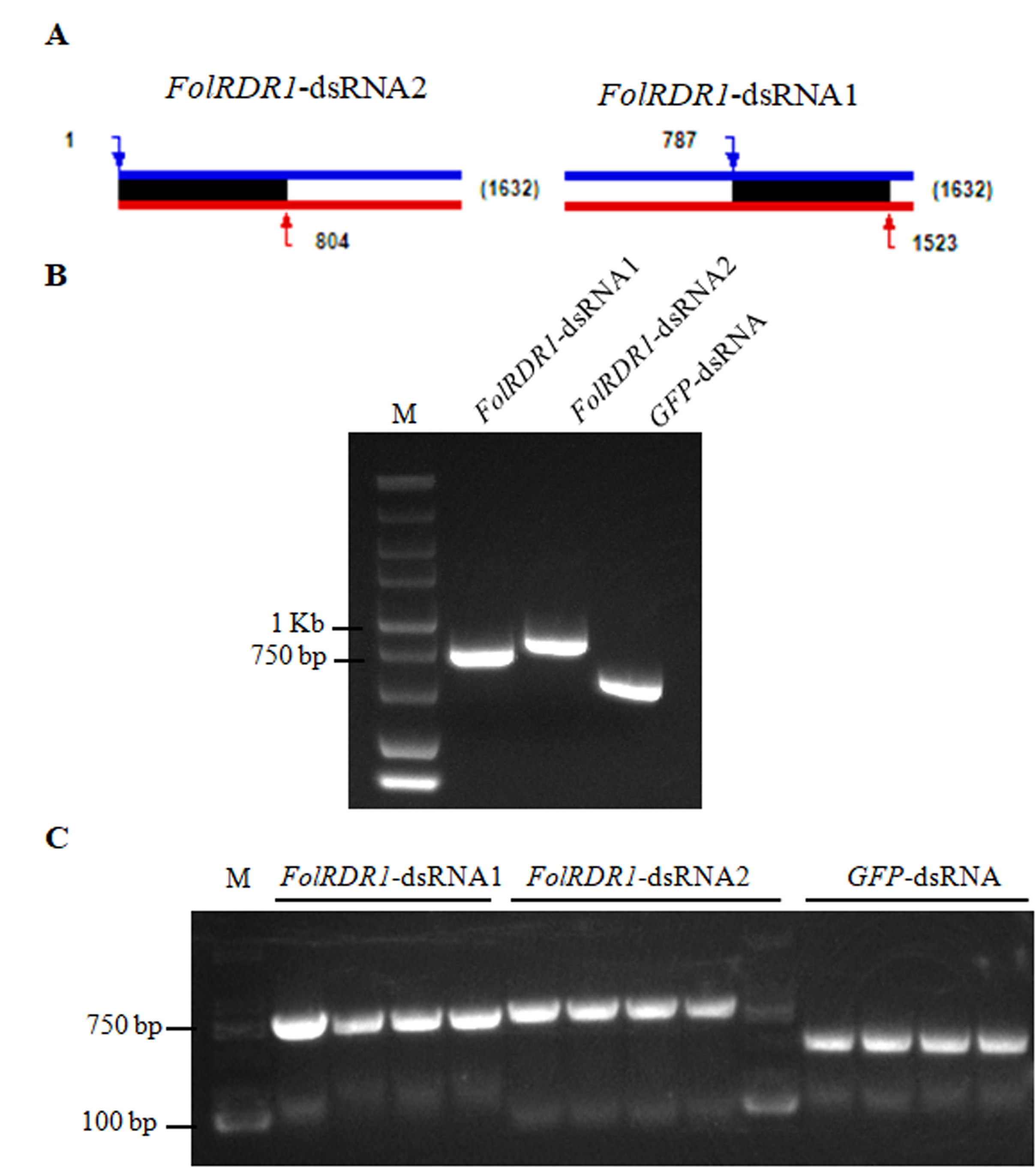

Supplement: S7 Fig — A Sketch map of FolRDR1-dsRNA1 and FolRDR1-dsRNA2. B Fragments of FolRDR1-dsRNA1, FolRDR1-dsRNA2 and GFP-dsRNA were amplified using gene-specific primers. C Diagnostic PCR was used to identify positive clones. (TIF) [file ppat.1011463.s007.tif]

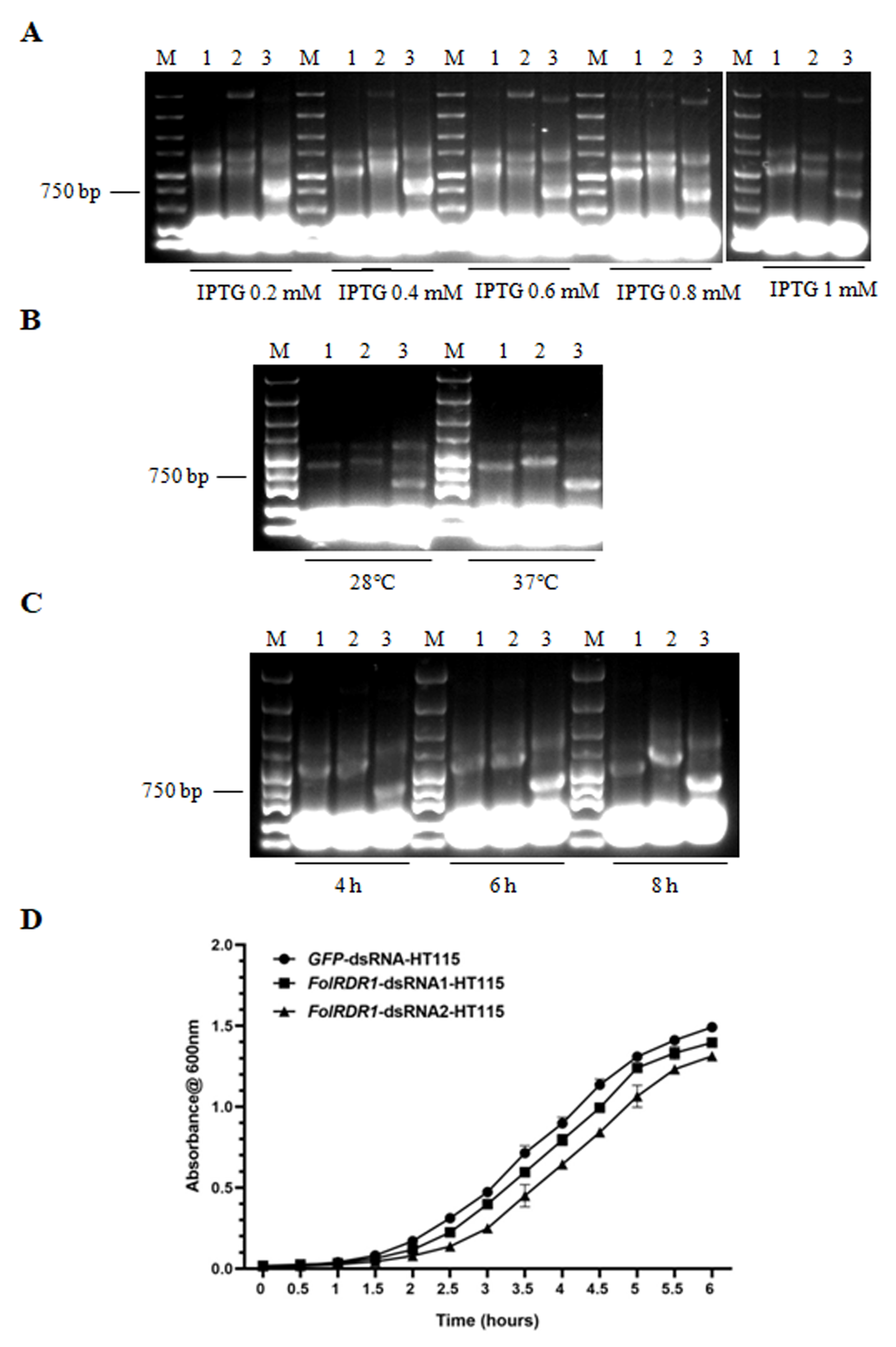

Supplement: S8 Fig — A Expression strains of FolRDR1-dsRNA1, FolRDR1-dsRNA2 and GFP-dsRNA were induced using different concentration of IPTG. B Abundance of FolRDR1-dsRNA1, FolRDR1-dsRNA2 and GFP-dsRNA were scaled under different temperature. C Abundance of FolRDR1-dsRNA1, FolRDR1-dsRNA2 and GFP-dsRNA were scaled under different induced time points. D The growth curve of different strains measured by light transmittance (OD = 600 nm). a presents no significant differences (p>0.05), d, e present significant differences (P<0.01). (TIF) [file ppat.1011463.s008.tif]

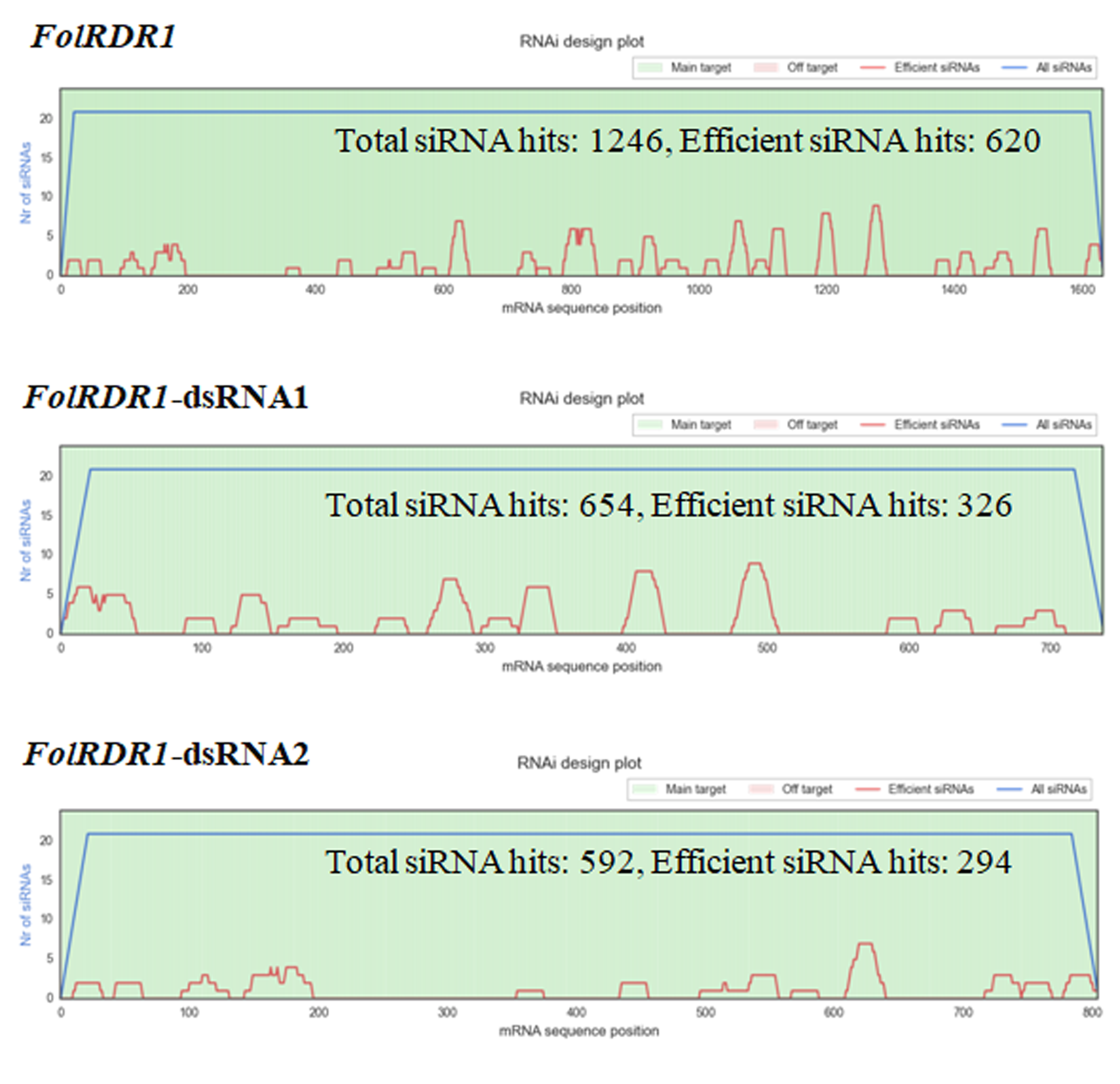

Supplement: S9 Fig — Efficient siRNAs generated in different regions of FolRDR1 were predicted using siRNA-Finder (Si-Fi). Briefly, the off-target searching pipeline starts with splitting a long RNAi trigger sequence (the complement to the target sequence of the corresponding RNA) into all possible MERs using stringent parameters (stricter strand selection rules plus target site accessibility calculations). (TIF) [file ppat.1011463.s009.tif]

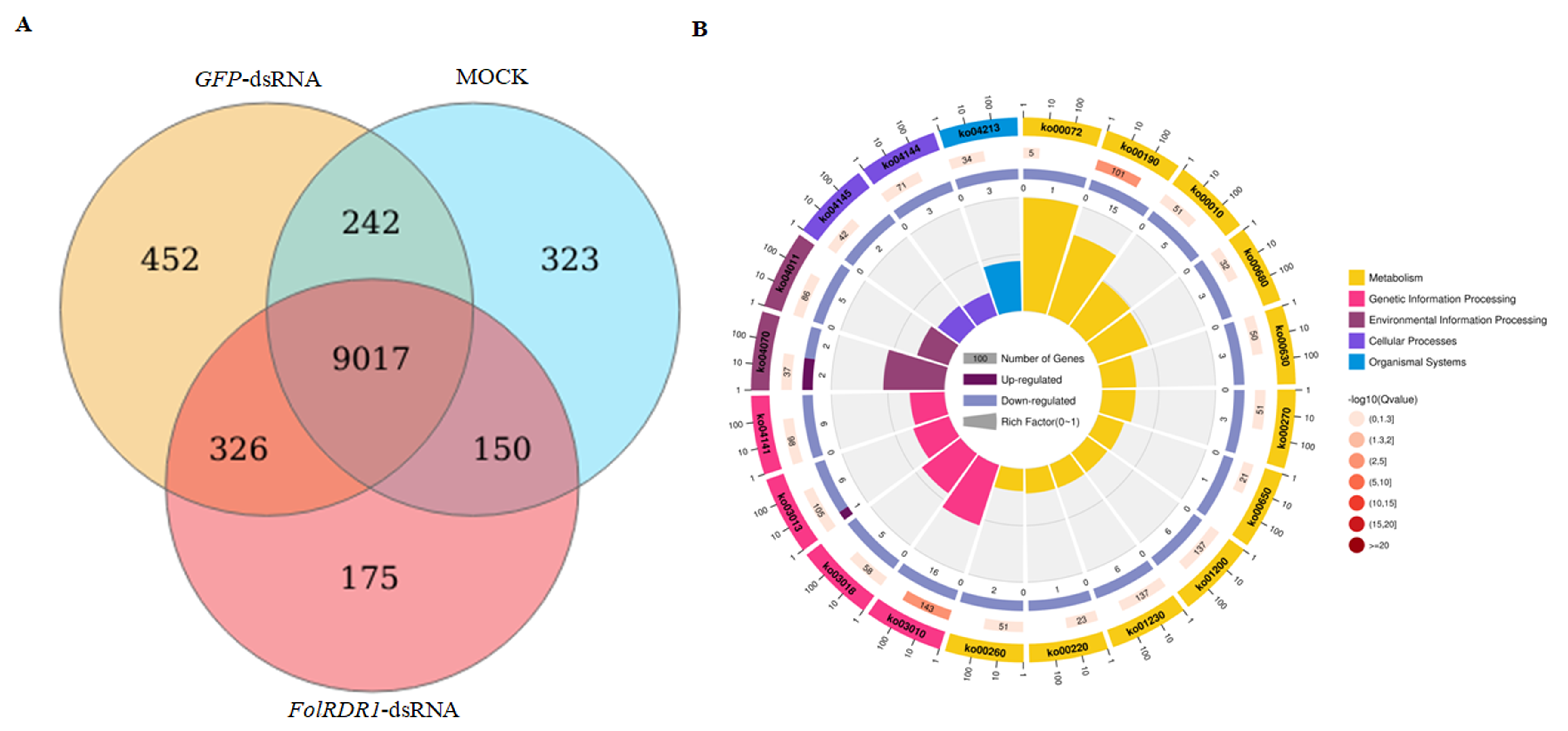

Supplement: S10 Fig — The samples were collected at 24 hours after treatment. A The number of DEGs (Different Expressed Gene) in RNA-seq libraries using water (Mock), GFP-dsRNA (negative control), FolRDR1-dsRNA1. B Analysis of KEGG (Kyoto Encyclopedia of Genes and Genomes). (TIF) [file ppat.1011463.s010.tif]

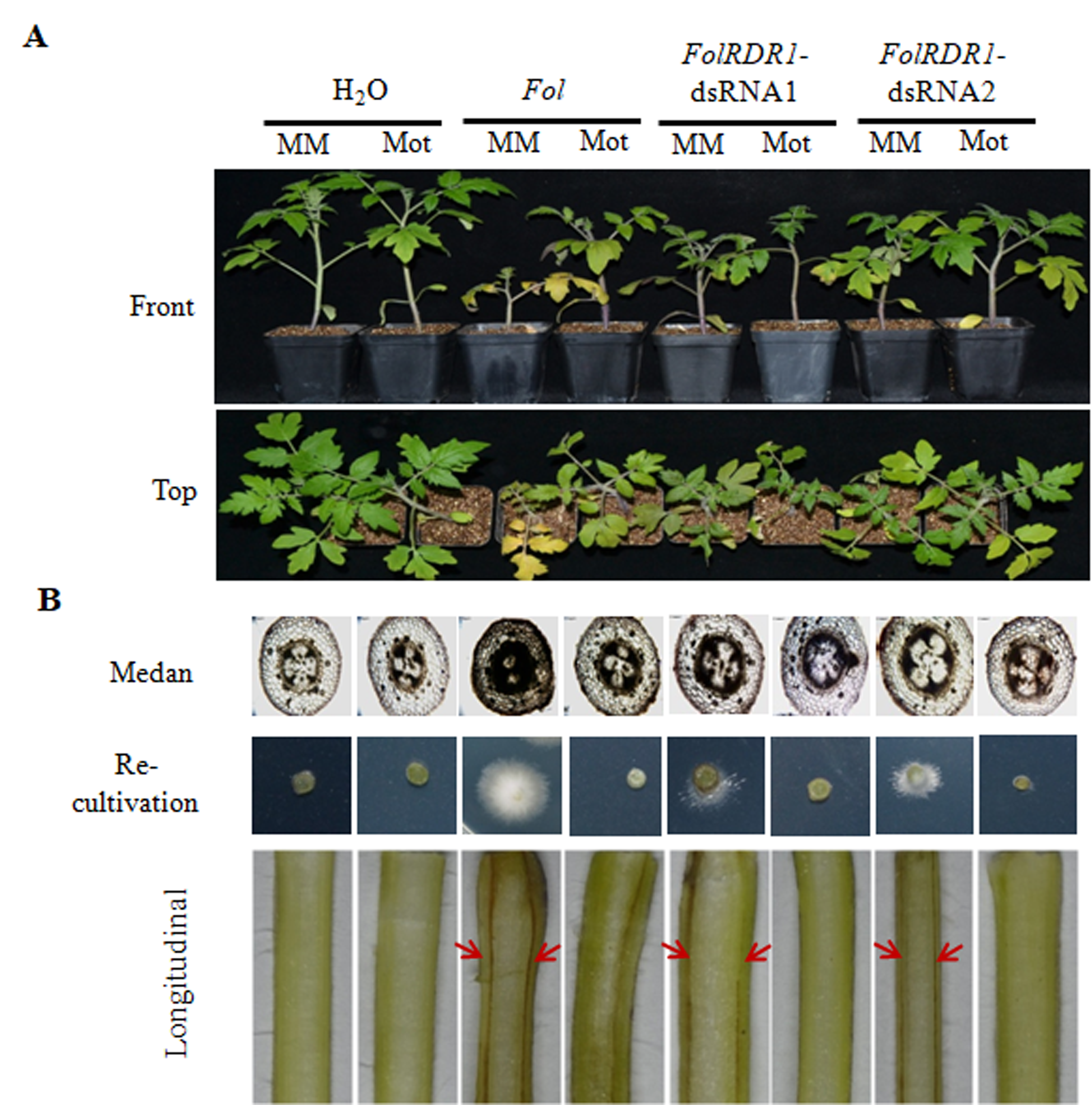

Supplement: S11 Fig — A Two-week tomato seedlings were sprayed with FolRDR1-dsRNAs on the leaves respectively, followed by infecting by WT Fol two days later as described previously. Wilt disease symptoms were photographed 2 weeks after inoculation. Front, images were taken from the front of plants. Top, images were taken from the top of plants. B Cotton blue staining results reflect the abundance of Fol in the stem of tomato plants. More intense cotton blue staining correlates with higher levels of Fol (Up panel). The outgrowth of fungi from tomato stems of plants inoculated with the indicated strains on PDA, and images were taken at 2 dpi, respectively (Middle panel). Diseased vascular bundles were checked in longitudinal splitting stem (Pointed by red arrows) (Bottom panel). (TIF) [file ppat.1011463.s011.tif]
