# Supplementary material for: Artificial trans-kingdom RNAi of FolRDR1 is a potential strategy to control tomato wilt disease
Source: PLoS Pathog. 2023 Jun 20;19(6):e1011463. doi: 10.1371/journal.ppat.1011463 (PMC10313012; doi:10.1371/journal.ppat.1011463)
Supplement: S1 Table — (DOC) [file ppat.1011463.s012.doc]

**Table S1** Primers used in this study.

| Purpose | ID | Sequence（5’→3’） |
| --- | --- | --- |
| Gene cloning | *Fol*RdsR1-2F | GGCCTGCATTGTGAACCATC |
|  | *Fol*RdsR1-2R | GTCTCATCTCGCGTCTCTGG |
|  | *Fol*RdsR5-2F | ATGTCCACTTCTCAAGGCCG |
|  | *Fol*RdsR5-2R | TGGTTCACAATGCAGGCCA |
|  | *GFP*dsR1-2F | TTCCATGGCCAACACTTGTC |
|  | *GFP*dsR1-2R | CGAAAGGGCAGATTGTGTGG |
|  | *GFP*-Xba I-F | TGCCTAGATTCCATGGCCAACACTTGTC |
|  | *GFP*-Hind III-R | CCCAGCTTCGAAAGGGCAGATTGTGTGG |
|  | T7-*Fol*Rds-1F | TAATACGACTCACTATAGGGGGCCTGCATTGTGAACCATC |
|  | T7-*Fol*Rds-1R | TAATACGACTCACTATAGGGGTCTCATCTCGCGTCTCTGG |
|  | T7-*Fol*Rds-5F | TAATACGACTCACTATAGGGATGTCCACTTCTCAAGGCCG |
|  | T7-*Fol*Rds-5R | TAATACGACTCACTATAGGGTGGTTCACAATGCAGGCCA |
|  | T7-*GFP*dsR1-2F | TAATACGACTCACTATAGGGTTCCATGGCCAACACTTGTC |
|  | T7-*GFP*dsR1-2R | TAATACGACTCACTATAGGGCGAAAGGGCAGATTGTGTGG |
|  | *Fol*RhpR51-F | CATGCCATGGCATGATGTCCACTTCTCAAGGCCG |
|  | *Fol*RhpR52-R | CCCAAGCTTGGGTCCATATGTTGAGGTGCTCG |
|  | *Fol*RC-F | ATGTCCACTTCTCAAGGCCG |
|  | *Fol*RC-R | TCAAAACATGTTCGGGAAGAG |
|  | *Fol*RdsR-1F | GTCGACGGTATCGATAAGCTTGGCCTGCATTGTGAACCATC |
|  | *Fol*RdsR-1R | GGCGGCCGCTCTAGAACTAGTGTCTCATCTCGCGTCTCTGG |
|  | *Fol*RdsR-5F | GTCGACGGTATCGATAAGCTTATGTCCACTTCTCAAGGCCG |
|  | *Fol*RdsR-5R | GGCGGCCGCTCTAGAACTAGTTGGTTCACAATGCAGGCCA |
|  | *GFP*dsR-F | GTCGACGGTATCGATAAGCTTTCTGTCAGTGGAGAGGGTGA |
|  | *GFP*dsR-R | GGCGGCCGCTCTAGAACTAGTTGGTCTGCTAGTTGAACGCT |
| Diagnostic | M13-F | GTAAAACGACGGCCAG |
|  | M13-R | CAGGAAACAGCTATGAC |
|  | Test-*GFP*-F | TTCCATGGCCAACACTTGTC |
|  | Test-*GFP*-R | CGAAAGGGCAGATTGTGTGG |
|  | T7 | TAATACGACTCACTATAGGG |
| qRT-PCR | *Fol*-18s rRNA-F | CGCCAGAGGACCCCTAAAC |
|  | *Fol*-18s-Rrna-R | ATCGATGCCAGAACCAAGAGA |
|  | Oligo-dT | TTTTTTTTTTTTTTTTTT |
|  | *Fol*Rq-1F | GCTAGTGTCACAGGTTGGCT |
|  | *Fol*Rq-1R | GGATGGTTCACAATGCAGGC |
|  | *Fol*Rq-2F | ACTCTGCTTCCTTCACCTCC |
|  | *Fol*Rq-2R | CTAAGAAACGCAGCCCCAGA |
|  | *Fol*Acq-1F | GTCACCAACTGGGACGACAT |
|  | *Fol*Acq-1R | GGACTTGGGGTTGATGGGAG |
|  | *Fol*Acq-2F | TTCCCTTCCATTGTCGGTCG |
|  | *Fol*Acq-2R | TGGGGTATCGCAGAGTGAGA |
|  | *Fol*-IGS1049 | TGCGATTTGGACGAGATATGTG |
|  | *Fol*-IGS1050 | ATTTGCCTACCCTGTACCTACC |
|  | *Sly*-18s rRNA-F | TGACGGAGAATTAGGGTTCG |
|  | *Sly*-18s rRNA-R | CCTCCAATGGATCCTCGTTA |
