# Supplementary material for: Artificial trans-kingdom RNAi of FolRDR1 is a potential strategy to control tomato wilt disease
Source: PLoS Pathog. 2023 Jun 20;19(6):e1011463. doi: 10.1371/journal.ppat.1011463 (PMC10313012; doi:10.1371/journal.ppat.1011463)
Supplement: S4 Table — (DOC) [file ppat.1011463.s015.doc]

**Table S4** Prediction of *FolRDR1* off-target transcripts. Simulations were run using Si-Fi software (v3.1) for off-target prediction ( http://labtools.ipk-gatersleben.de ); Gene sequence files of different organisms from NCBI Genome Assembly/Annotation Projects.

| **Species** | **Reference genome** | **Targets** |
| --- | --- | --- |
| *Fusarium oxysporum* | Fusarium oxysporum NRRL 32931 (assembly FO_FOSC_3_a_V1) | 1 |
| *Arabidopsis thaliana* | Arabidopsis thaliana (assembly TAIR10) | 1 |
| *Nicotiana tabacum* | Nicotiana tabacum (assembly Ntab-TN90) | 3 |
| *Triticum aestivum* | Wheat Pangenome v1.0 Assembly | 1 |
| *Oryza sativa* | Oryza sativa Japonica Group (assembly IRGSP-1.0) | 2 |
| *Solanum lycopersicum* | Solanum lycopersicum (assembly SL3.0) | 0 |
| *Arachis hypogaea* | Arachis hypogaea (assembly arahy.Tifrunner.gnm1.KYV3) | 0 |
| *Glycine max* | Glycine max (assembly Glycine_max_v4.0) | 0 |
| *Zea mays* | Zea mays (assembly Zm-B73-REFERENCE-NAM-5.0) | 0 |
| *Raphanus sativus* | Raphanus sativus (assembly Rs1.0) | 2 |
| *Brassica campestris* | Brassica rapa (assembly CAAS_Brap_v3.01) | 0 |
| *Cucumis sativus* | Cucumis sativus (assembly Cucumber_9930_V3) | 0 |
| *Cucurbita moschata* | Cucurbita moschata (assembly Cmos_1.0) | 0 |
| *Botrytis cinerea* | Botrytis cinerea B05.10 (assembly ASM14353v4) | 0 |
| *Pleurotus eryngii* | Pleurotus eryngii (assembly ASM324376v1) | 0 |
| *Drosophila melanogaster* | Drosophila melanogaster (assembly Release 6 plus ISO1 MT) | 0 |
| *Caenorhabditis elegans* | Caenorhabditis elegans (assembly WBcel235) | 0 |
