# Supplementary material for: Artificial trans-kingdom RNAi of FolRDR1 is a potential strategy to control tomato wilt disease
Source: PLoS Pathog. 2023 Jun 20;19(6):e1011463. doi: 10.1371/journal.ppat.1011463 (PMC10313012; doi:10.1371/journal.ppat.1011463)
Supplement: S5 Table — (DOC) [file ppat.1011463.s016.doc]

**Table S4** Sequences of DNA probes

| **ID** | **Sequence (5’ – 3’)** | | **Location in CDS (nt)** |
| --- | --- | --- | --- |
| Probe 1 | CTGTGTCCGTTTTGAGTATGACTGCACCTA | 96 – 125 | |
| Probe 2 | ATGACGCTACTCTGCTTCCTTCACCTCCTG | 158 - 187 | |
| Probe 3 | AGACCATACAAGGTAGATGGGCTGGTTATA | 500 - 529 | |
| Probe 4 | ACCAGTGGAGCTTGACTTAATAGAAACAGC | 606 - 635 | |
| Probe 5 | GTCGTATATCTGAGAATAGCTGGAACACCT | 706 - 735 | |
| Probe 6 | AGTCCTTCCAGTAACAGAAGAAAAGGTCGA | 816 - 845 | |
| Probe 7 | GAAGGAGACTATACCCATGAGCTCATGGAG | 967 - 996 | |
| Probe 8 | ATGTCTCTGCCGTCGACTTCAATCTATGAA | 1131 - 1160 | |
| Probe 9 | TTAGATGCTCGCTCGCCATGGCATCACATG | 1237 - 1266 | |
| Probe 10 | CTTATACTGATGCAACAGAGGCGCAAGGAG | 1420 - 1449 | |
