# Supplementary material for: Artificial trans-kingdom RNAi of FolRDR1 is a potential strategy to control tomato wilt disease
Source: PLoS Pathog. 2023 Jun 20;19(6):e1011463. doi: 10.1371/journal.ppat.1011463 (PMC10313012; doi:10.1371/journal.ppat.1011463)
Supplement: Attachment — Submitted filename: Response to comments-PPATHOGENS-D-22-02186.pdf [file ppat.1011463.s018.pdf]

Responses to comments

Reviewer #1:

-All main figures – all pictures are in poor quality and it's really difficult to judge the plate and plant phenotypes since it is all blurry.

Response: Thanks for this comment. All pictures were improved with appropriate resolution.

-The choice of RDR1 as the silencing target in Fol is very peculiar and not well explained. Why would the authors want to silence a component of the cell silencing machinery? Wouldn't it hurt the efficiency of the silencing itself? Why was this gene silenced and not other housekeeping gene that is required for survival? In addition, the characterization of the rdr1 mutants appear to be detached from the putative cellular function of this gene. It will be more appropriate to check whether gene silencing is affected in this mutant as well.

Response: We appreciate for these pertinent comments. This project was initiated in 2019, when the genome information of *Fusarium oxysporum* f.sp. *Lycopersici* (*Fol*) wasn't detailed and accurate (actually, it still needs to be greatly improved currently). Our group is consistently engaged in the study of the molecular mechanism of fusarium wilt disease resistance in tomato, especially focuses on sRNA function. We tried to find the genes, such as *DCL*, *RDR*, *AGO* and other gene families, related to the production and function of sRNA that might exist in the genome of *Fol* by homologous comparison. However, with the existing gene annotation of *Fol*, only *FolRDR1* was identified. We know that the selection of *FolRDR1* as the research object is debatable from the perspective of raising scientific issues, but our experimental results show that *RDR1* is indeed closely related to the pathogenicity of *Fol*. We have made some necessary explanations about this in the Introduction section.

To assess the potential regulated gene by impaired *FolRDR1*, the miRNA levels were evaluated by sRNA-seq using the KO-strains *FolRDR1*-KO-36 (named as *FolRDR1*-1 in library), *FolRDR1*-KO-126 (named as *FolRDR1*-2 in library) and wild type strain (named as *Fol*-WT in library). The DEGs of miRNAs were listed in updated Table S2. Well correlation was showed between *FolRDR1*-KO-36 and *FolRDR1*-KO-126 (Results updated in the text as Fig S3A). Compared with WT strain, the abundances of miRNAs declined significantly in both *FolRDR1*-KO strains (Results updated in the text as Fig S3B,C). We further analyzed the biological functions of predicted targets of miRNAs (Listed in updated Table S3), and the results showed that knock-outing of *FolRDR1* mainly affected the metabolic pathway in both KO strains (Results updated in the text as Fig S4). With above results, we concluded that the levels of miRNA was correlate with *FolRDR1* in *Fol*.

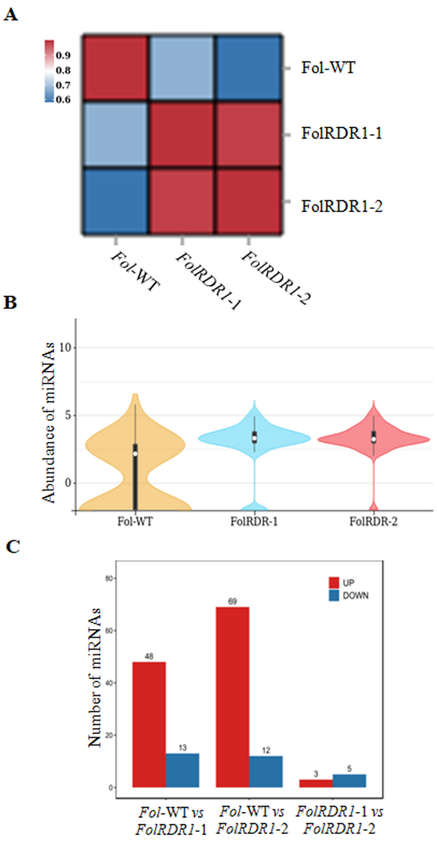

Figure S3 The analysis of sRNA-seq using *FolRDR1*-KO and WT strains. In the library, KO-strains *FolRDR1*-KO-36 was named as *FolRDR1*-1, *FolRDR1*-KO-126 was named as *FolRDR1*-2, and wild type strain was named as *Fol*-WT. **A** Correlation heat map analysis. **B, C** The abundances of miRNAs declined in both *FolRDR1*-KO strains.

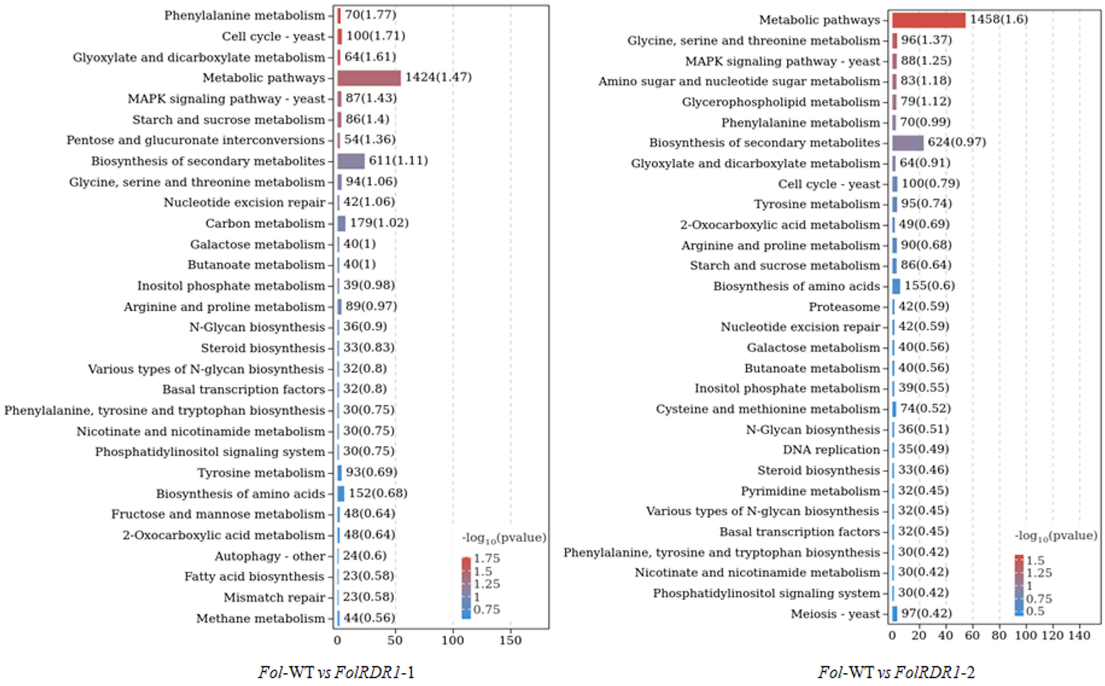

Figure S4 The analysis of GO. Knockouting of *FolRDR1* mainly affected the metabolic pathway in both KO strains.

-Figure 6D – According to this figure the authors showed that the amount of Fol in Fol-infected plants was only three folds of the mock infected plants (that are not supposed to contain any Fol at all!!!) which seems unreasonable. How specific are the IGS primers? Is it possible that they are also amplifying other fungal endophytes in the plant? Can the authors repeat this experiments with a different set of primers?

Response: Thanks for comments. The IGS primers of *Fol* used in this study were published in several articles previously, and the specificity of these primers is out of problem in our lab. As required, two more primer pairs were selected from the IGS region of *Fol* (The sequence of IGS was presented below). All three primer pairs (sequences were underlined) were used to assess the abundance of *Fol* biomass in tomato tissues. The results showed the specificity of these primers. Due to the length of the article, we only put the PCR results using *Fol*-IGS-F1/R1 in Fig 6C, meanwhile, Fig 6D was updated.

>AY118124.1 *Fusarium oxysporum* f. sp. *lycopersici* MM66 large subunit ribosomal RNA gene and large subunit ribosomal RNA-small subunit ribosomal RNA intergenic spacer region (IGS), partial sequence

TACCACCCGCACGTATAGATGGACAAGAATAGGCTTCGGCTTAGCGTCTTAGCAGGC
GATTCTTCCACGGCGCTCGAAGCGCGTCGTGGTATTTCGCGTATTGTAATTTCAACAC
GAGCGGGGTCAAATCCTTTGCAGACGACTTAGCTGTGCGAAACGGTCCTGTAAGCAG
TAGAGTAGCCTTGTTGTTACGATCTGCTGAGGGTAAGCCGTCCTTCGCCTCGATTTCC
CCAATGGGTTCTCCGGATTTCTGGAGACTTGTAGGGGTTGTGGGATTTTTGATGTGTC
GTCTCCGGACGGGCGGTGCAGGGTAGTCGAGTTAGACTTGGTGGAATTCCGTCGATA
GGAGTTCCGTCGAGTCTGGTCAGCTGTGTGTTGGACGGTGTAGGGTAGGCTGCTTGGA
CATGGTCGGTTCGAGGATCGATTCGAGGGCCGGCCTGTCGATGATGTGTGATGTATGC
GGTCTAGGGTAGGTTGGTTTGTCTTGGTTCGATTTGATGTCGGCTCCCGTGCAGGCCA
GAGTGAGGGGGGTCCAGGGTAGGTGCAGGGTAGGCAGCTTAGATTTGGTCGATCTGG
AGGTCGATTCTCCGGCTGGCGGATCTGACACTGTCGAAACGAGATGCGAGGGGTGTA
GGGTAGGCTAGTTTCGTACTTGCCAGGTTGCGATTTGGACGAGATATGTGGTTTAGGG
TAGGCTCTAGGGTAAGTAGAATTCGAGTTTCGTCGCCGATAGTTTTCTGTGGGTGTAT
GGTAGGTACAGGGTAGGCAAATCTCTCTCCGGCCAGTACTTGTCTCGTGGTCGTGAGT
CGATTTTTTTGTTTTGCCATACTATTGAATTTTGCGGAAATTCAAAAGTGGCTCGGGA
GTCCCGCCTGGCGTGCGTCCGACTCGAACATCGTCGGTGTACATATGAGAGGGAGTA
ATCCGGCCCGGCCGGGGCCCATCGCGAGCTGCCGGGTAGGTAAAAGT

Fol-IGS-F1	TGCGATTTGGACGAGATATGTG
Fol-IGS-R1	ATTTGCCTACCCTGTACCTACC
Fol-IGS-F2	CGGTCCTGTAAGCAGTAGAG
Fol-IGS-R2	CAACCCCTACAAGTCTCCAG
Fol-IGS-F3	GGGATTTTTGATGTGTCGTC
Fol-IGS-R3	CTACACCGTCCAACACACAG

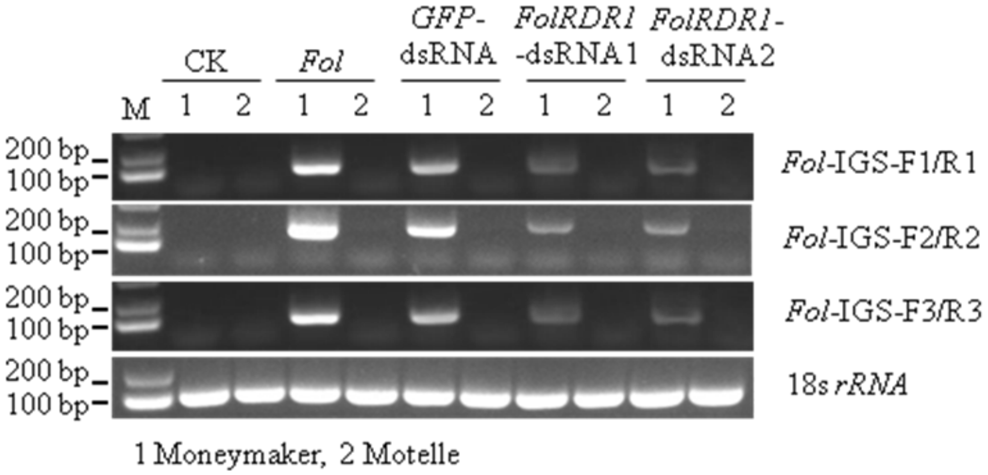

-Figure 5 – the presence of the sdRNA in the stem and the root should be monitored by RT-PCR or qRT-PCR as well.

Response: Thanks for comments. The required data were updated in Fig 5A.

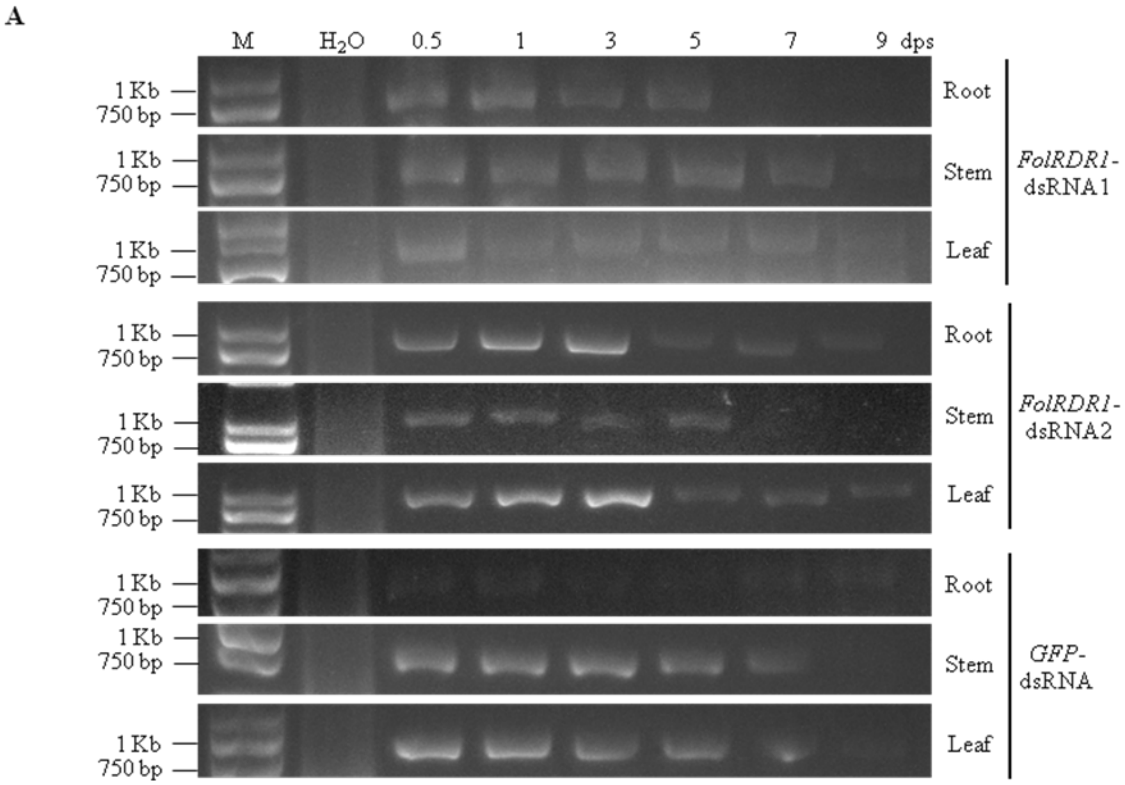

Reviewer #2:

• In the paper, the authors claim that the application of FolRDR1-dsRNAs on the surface of pre-infected tomato seedling leaves significantly inhibited the development of Fusarium wilt. Since FolRDR1-RNAi-SIGS works like a bio-pesticide, what are results of applying FolRDR1-dsRNAs before Fol infection? Whether it can provide better protection in the lab?

Response: Thanks for this constructive comment. Actually, we performed both pre-treatment and after-treatment with *FolRDR1*-dsRNAs experiments. The data indicated that no significant difference was shown between these two treatments. Due to the unpredictable development of fusarium wilt disease in field, we think that application in the early stage of disease might reduce the cost of disease control. Therefore, only after-treatment with *FolRDR1*-dsRNAs experiment was presented in this manuscript.

Reviewer #3:

1. The knockout experiments indicated that the FolRDR1 is required for the vegetative growth and asexual reproduction in Fol (Figure 1-2). As the authors mentioned that RDRs interact with RNAi machinery and sense transgene-induced silencing to generate dsRNA molecules in fungi (Line 95-103, 266-269), whether the function of RNA silencing pathway and accumulation of sRNA was affected in the FolRDR1-KO strains? Koch et al. (2016) demonstrated that the F. graminearum DCL1 protein is required for exogenous dsRNA processing and intact fungal silencing machinery was required for SIGS in F. graminearum. If the FolRDR1 is essential for sRNA accumulation, when FolRDR1 was silenced by exogenous FolRDR1-dsRNA, this would in turn interfere with the own RNAi process of Fol. In order to ensure the efficiency of SIGS, the function of dsRNA targeted genes in the pathogen RNAi silencing process should be carefully considered. I suggest that small RNA sequencing analysis or northern blot should be carried out to explore the function of FolRDR1 and confirm the intact RNAi machinery of the FolRDR1-KO strains.

Response: Thanks for comments. As required, small RNA sequencing analysis was performed using the KO-strains *FolRDR1*-KO-36 (named as *FolRDR1*-1 in library), *FolRDR1*-KO-126 (named as *FolRDR1*-2 in library) and wild type strain (named as *Fol*-WT in library). The DEGs of miRNAs were listed in updated Table S2. Well correlation was showed between *FolRDR1*-KO-36 and *FolRDR1*-KO-126 (Results updated in the text as Fig S3A). Compared with WT strain, the abundances of miRNAs declined in both *FolRDR1*-KO strains (Results updated in the text as Fig S3B,C). We further analyzed the biological functions of predicted targets of miRNAs (Listed in updated Table S3), and the results showed that knockouting of *FolRDR1* mainly affected the metabolic pathway in both KO strains (Results updated in the text as Fig S4). With above results, we concluded that the levels of miRNA were correlated with *FolRDR1* in *Fol*.

2. Line 120-125. The authors found that FolRDR1 was highly conservative in different F. oxysporum races, it is necessary to understand its conservation across species for a functional important protein. And the authors should make clear that how many RDR genes are present in the

genome of Fol, and why FolRDR1 was chose for knockout.

Response: Thanks for this comments. When this project was initiated in 2019, we tried to find the genes related to the production and function of small RNA, such as *DCL*, *RDR*, *AGO* and other gene families that might exist in the genome of *Fol*. However, with the existing gene annotation of *Fol*, only *RDR1* was predicted both in NCBI and JGI (The Fungal Genomics Resource, https://mycocosm.jgi.doe.gov/Fusox2/Fusox2.home.html) in *Fol* genome, but not studied yet. We know that the selection of *RDR1* as the research object is debatable from the perspective of raising scientific issues, but our experimental results show that *RDR1* is indeed closely related to the pathogenicity of *Fol*.

3. The authors proved that artificial FolRDR1-dsRNAs can be taken up by Fol, and the mRNA level of RDR1 is repressed (Fig3). I suggest using Northern blot to detect the corresponding RDR1-sRNAs accumulated in the fungal mycelia to demonstrate that the dsRNA were processed into sRNAs in Fol.

Response: Thanks for this comments. As required, the corresponding levels of *Fol*RDR1 in the fungal mycelia were checked using Northern blot, and this data was updated as Fig 3E.

E

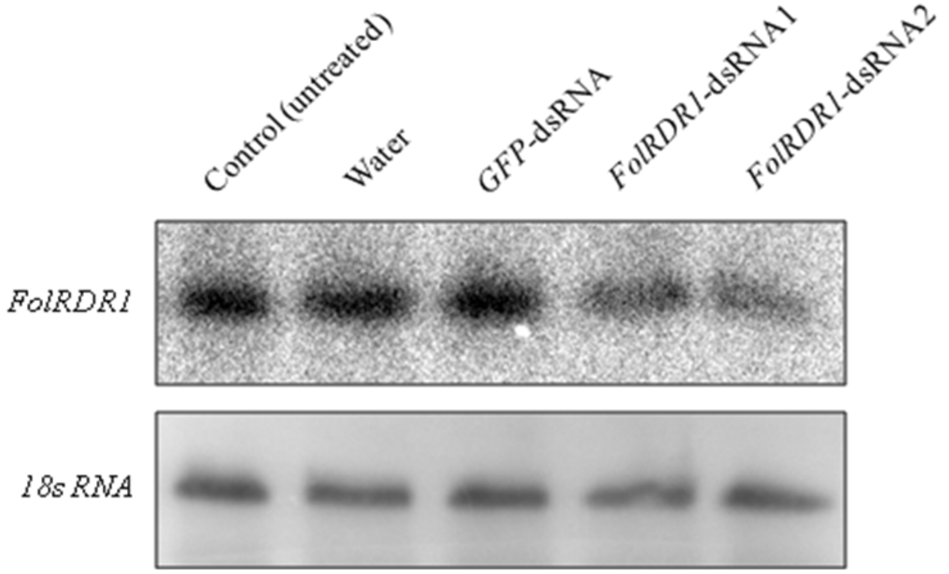

4. The fluoresce signals observed in the undaubed leaves were weak and uneven in fig5C, the brighter spots look like autofluorescence from damaged leave cells. The quality of the pictures should be improved. In the stem vascular tissue (Fig5D), whether fluorescence signals in phloem and xylem can be distinguished with higher magnification. In addition, why the fluoresce signals were rich in the root hair in fig5E? And most importantly, whether the FolRDR1-sRNAs accumulated in tomato roots after taking up the FolRDR1-dsRNAs from leaves?

Response: Thanks for this comment. In this experiment, all images were taken from live leaf samples which were thick and big to microscope lens. Due to the limited resolution, we tried our best to collected current images, especially for undaubed leaves. Our updated results showed that dsRNAs were transferred from treated leaf to root (updated in Fig 5A), and we speculated this process is fulfilled via vascular bundle.

5. Spray of FolRDR1-dsRNAs alleviated the development of tomato wilt disease in fig6. However, the authors just observed the development of tomato wilt disease, did not monitor the accumulation of the FolRDR1-sRNAs in the infected tomato, neither detect the transcript level of FolRDR1 gene during infection. And I think it's important to verify that the FolRDR1-dsRNAs or the FolRDR1-sRNAs were taken up by the Fol, and processed in the mycelia to induce the RNAi in the pathogen. Only then can it be termed cross-kingdom RNAi.
Response: Thanks for this comment. The accumulation of the *FolRDR1*-sRNAs in the infected tomato tissues including leaf, stem and root were monitored which was updated in Fig 5A as required. The results of the transcript level of *FolRDR1* during infection were presented as Fig 2B. Our data showed that both *FolRDR1*-dsRNAs and *GFP*-dsRNAs were taken up by *Fol* (Fig 3A). Further, the results Fig 3C indicated that *FolRDR1*-dsRNAs and *GFP*-dsRNAs were processed in the mycelia.

6. Many results lack experimental support in the Materials and methods section and the experimental conditions are not well described. Some examples, (1) Line 170, 179, 210, 230, 376-377, and 380-382, the concentration or the amount of the dsRNAs used? (2) Line 198-202, what's the experimental conditions set to explore the optimal treatment in fig4D and fig4E. (3) How long the Fol took to produce conidia? And how to assess the production of the conidia in fig1D and fig4C? (4) Line 229-231, How long a gap should take to spray the dsRNA after inoculation of Fol? (5) For all the inoculation experiments, how many tomato seedlings were inoculated in one experiment, there was no statistics on the severity of the disease in fig 2C and fig6.

Response: (1) For these treatments, the concentration of both *FolRDR1*-dsRNAs and *GFP*-dsRNAs was 150 ng/mL. We detailed accordingly in the main text. (2) In this experiment, we designed different concentration of dsRNA and time of treatment to optimize the treatment condition. Finally, the optimal conditions with concentration of 150 ng/mL and treating for 24 hours in liquid PDA medium were used for further experiments. (3) To our knowledge, conidia could be observed in 24 hours in liquid medium. "Statistics of spore production" was added in the Materials and methods. (4) To assess whether SIGS attenuate Fol infection, we sprayed the *Fol* pre-treated 2-week tomato seedling with FolRDR1-dsRNAs (200 ng/mL) at 24 hours after infecting with *Fol* and scaled the development of Fusarium wilt symptoms. We revised accordingly in the main text. (5) For all the inoculation experiments, at least 30 tomato seedlings were inoculated for each treatment. The statistical analysis was performed as required.

Part III – Minor Issues: Editorial and Data Presentation Modifications
Please use this section for editorial suggestions as well as relatively minor modifications of existing data that would enhance clarity.

Reviewer #1:

-lines 227-255/figure 6 – how long after dsRNA treatment were the plants infected with Fol?
Response: To assess whether SIGS attenuate *Fol* infection, we sprayed the *Fol* pre-treated

2-week tomato seedling with *FolRDR1*-dsRNAs (200 ng/mL) at 24 hours after infecting with *Fol* and scaled the development of Fusarium wilt symptoms. We updated the details in the main text.

-How long after spraying with dsRNA were the leaves collected for RNA-seq analysis? dsRNA was previously suspected to act as an immune elicitor. The fact that neither dsGFP nor dsRDR1 caused any significant transcriptional changes in the plant is a bit surprising, unless the samples were collected long after spray application.
Response: The samples were collected at 24 hours after treatment, and we updated the details in the legend of Fig S10.

We agree with that dsRNA is previously suspected to act as an immune elicitor. In this study, *FolRDR1*-dsRNAs were used to inhibit the pathogenicity of *Fol* in tomato plants. We speculate that neither ds*GFP*-dsRNA nor ds*RDR1* caused any significant transcriptional changes in the plant due to gene homology in different species.

-Please provide a table of all the DEGs which were identified in the RNAseq analyses and their fold change.
Response: A list of all expressed genes was supplied as Table S5.

-All figure legends – the number of times the experiments were repeated and the number of biological replicates in each experiment should be stated in the figure legends.
Response: We updated the figure legend as required.

-Fig 2C – please split this section into three: Fig 2C - tomato pictures, Fig 2D – stem staining, Fig 2E –outgrowth assay.
Response: Figure 2 was updated as required.

-Figure S1B – why tomato actin was used for the tree construction? Is it a mistake?
Response: We apologize for this mistake. We replaced with *Fusarium* redolens uncharacterized protein (BKA55DRAFT_560103), mRNA.

-Figure S2B – The top (WT) and bottom (mutants) bands in the left gel are too close together and it's hard to really distinguish the size difference. Please repeat the experiment the run the gel longer.
Response: Figure S2B was updated as required.

-Table S2 - Please change the table title from "Table S1" to "Table S2"
Response: The title of Table S1 was changed to Table S2 as required.

-Line 145 – sentence is not clear. Please rephrase it.
Response: We rephrased this entire paragraph in the main text.

-Line 212- remove the word "at"
Response: It was removed in the main text.

-Line 235-236 – please rephrase the sentence
Response: We rephrased this sentence in the main text.

-Line 285-286 - please rephrase the sentence

Response: We rephrased this sentence in the main text.

Reviewer #2:

1) Please label the statistical analysis results in Figures 4C, D, E, and 6D.

Response: We performed the statistical analysis as required.

2) In Figure 6A, the 15 d next to the bottom panel should be 25 d.

Response: We apologize for this mistake and fixed it.

3) Line 238-241, please analyze the interpret the data in Figure 6B-D.

Response: We rephrased this part in the main text.

4) Please add the catalog number for the agents used in work.

Response: The catalog numbers for the agents used in work were added accordingly.

5) Line 305 should be seedling leaves.

Response: We fixed this typo.

Reviewer #3:

1. Line 231-238, in the text the symptoms were described at 7 dpi and 25 dpi, while it was 15d in the figure 6A, and 2 weeks in the figure legend (Line 588). The descriptions must be consistent.
Response: We apologize for this mistake and fixed it.

2. FolRDR1-dsRNA1 and FolRDR1-dsRNA2 was designed as the first half and second half of the RDR1 gene respectively according to Fig S5, I suggest that the location of the qRT-PCR primers

should be labeled on the sequence to demonstrate the reliability of the transcription level of RDR1 detected by qRT-PCR.

Response: The sequences of primer *Fol*Rq-1F/R for *FolRDR1*-dsRNA1 and *Fol*Rq-2F/R for *FolRDR1*-dsRNA2 were presented in the Table S1.
