# Supplementary material for: Artificial trans-kingdom RNAi of FolRDR1 is a potential strategy to control tomato wilt disease
Source: PLoS Pathog. 2023 Jun 20;19(6):e1011463. doi: 10.1371/journal.ppat.1011463 (PMC10313012; doi:10.1371/journal.ppat.1011463)
Supplement: Attachment — Submitted filename: Response to comments-PPATHOGENS-D-22-02186-V2.pdf [file ppat.1011463.s019.pdf]

Reviewer's Responses to Questions

Part I - Summary

Please use this section to discuss strengths/weaknesses of study, novelty/significance, general execution and scholarship.

This study explores a new strategy to control tomato wilt disease based on RNAi. Exogenous application of *FolRDR1*-dsRNA effectively enhances the resistance of tomato seedlings to *Fusarium oxysporum* infection in lab conditions. Our data demonstrates that exogenous dsRNAs are absorbed and transported from sprayed leaves to other organs of the tomato plant through vascular bundle tissue, which highlights the consistence with the diffusion pathway of *Fusarium oxysporum* in the host plant. All results presented in this study rigorously support the key conclusions of this study, as well as provide a theoretical basis for developing new environment-friendly biological agents to control plant disease.

Fungal pathogens efficiently taking up environmental dsRNAs to induce the silencing of pathogen genes prompts the development of an innovative crop protection strategy, Spray-Induced Gene Silencing (SIGS) just several years ago. Here, we followed this direction to explore a new strategy for controlling tomato wilt disease. From a scientific perspective, innovation is limited. However, we expect our data will enrich the community of plant disease control.

Reviewer #1: The authors properly addressed all issues that were raised in the first review cycle.

The following small cosmetic adjustments should by added in the proof prior to publication:

*All figure legends - Please change "biological replicates in each…" to "biological replicates were used in each…"

Response: Thanks for this comments. We changed accordingly.

*Figures 6 and S8– the sentence "presents the largest average, de present significance…" is unclear. Please rephrase.

Response: Thanks for this comments. We rephrased accordingly.

*Figure S2C - "C" marking is missing. 1 kb and 2 kb markings in the DNA ladder were accidentally switched. Please fix it.

Response: We fixed it.

*Line 181 – please change "(Fig 2C)" to "(fig 2C-E)"

Response: We fixed it.

*Lines 240-251 – As requested, the authors also checked the presence of dsRNA in the stem and root by RT-PCR in addition to the labeled RNA (Fig 5A). This should be addressed in the text as well.

Response: We apologized for our careless and addressed accordingly.

Reviewer #2: The author mentioned that they performed both pre-treatment and after-treatment with FolRDR1-dsRNAs experiments and found that no significant difference was shown between these two treatments. Please show the data of pre-treatment with FolRDR1-dsRNAs experiment in the supplementary figures.

Response: As required, Fig S11 presenting the data of after-treatment with *FolRDR1*-dsRNAs experiment was added in the text. Two-week tomato seedlings were sprayed with FolRDR1-dsRNAs on the leaves respectively, followed by infecting by WT *Fol* two days later as described previously. Wilt disease symptoms were photographed 2 weeks after inoculation.

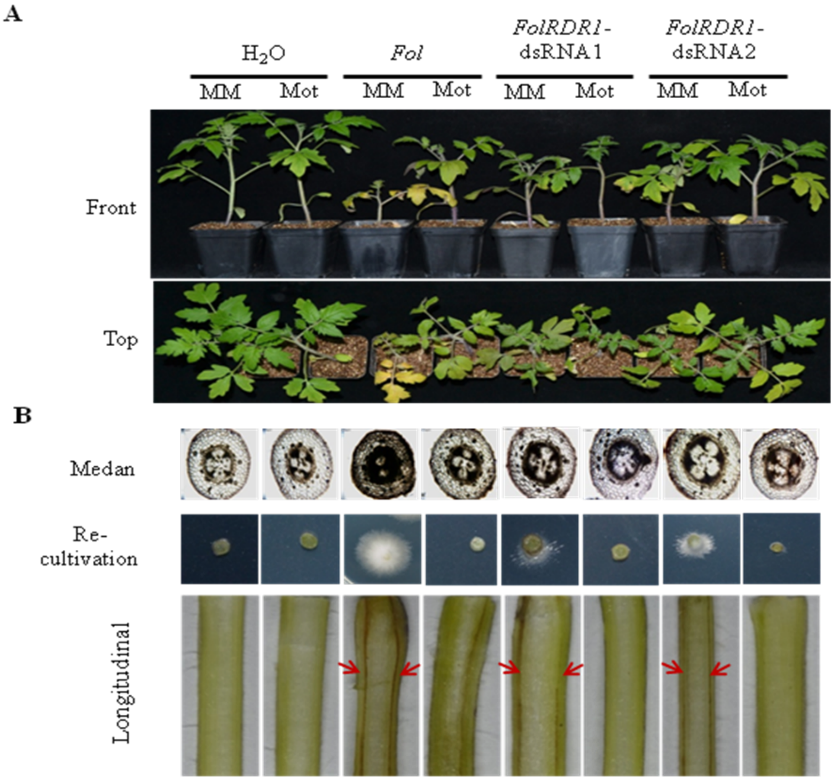

Reviewer #3: (No Response)

Part II – Major Issues: Key Experiments Required for Acceptance

Reviewer #1: (No Response)

Reviewer #2: (No Response)

Reviewer #3: To demonstrate the trans-kingdom RNAi in Fol, it is necessary not only to prove the RDR1-dsRNAs were taken up by Fol, but also that the dsRNAs were processed into small interfering (si)RNAs. The authors proved that artificial FolRDR1-dsRNAs can be taken up by Fol (Fig3) and tomato (Fig5) using confocal microscopy laser scanner and RT-PCR. But

there was no evidence of the accumulation of corresponding RDR1-dsRNA-derived siRNAs. Fig 3E and Fig 5A were updated to show the corresponding mRNA levels of FolRDR1 in the fungal mycelia and FolRDR1-dsRNAs transferred from treated leaves to stems and roots. However, these results did not prove that FolRDR1-dsRNAs were processed into siRNAs to induce RNAi in Fol. The authors may have misunderstood my questions. I suggested using Northern blot to detect the RDR1-dsRNA-derived siRNAs accumulated in the fungal mycelia (Fig3) and in the infected tomato (Fig6).

Response: Firstly, we apologize for our misunderstanding. To verify the production of *FolRDR1*-dsRNA-derived siRNAs, firstly, the efficient siRNAs generated in different regions of *FolRDR1* were analyzed using siRNA-Finder (Si-Fi) (Fig S9). Total ten 30-nt DNA fragments (detailed in Table S5), which uniformly distributed in the predicted regions in the CDS of FolRDR1, were synthesized and mixed as a pool followed by labeling with [γ-32P]ATP as probes for to detect the enrichment of *FolRDR1*-dsRNA-derived siRNAs using Northern blot. The data clearly shown that *FolRDR1*-dsRNA-derived siRNAs were enriched in both *FolRDR1*-dsRNA1 and *FolRDR1*-dsRNA2 treated fungal mycelia, respectively (Fig 3E, up panel).

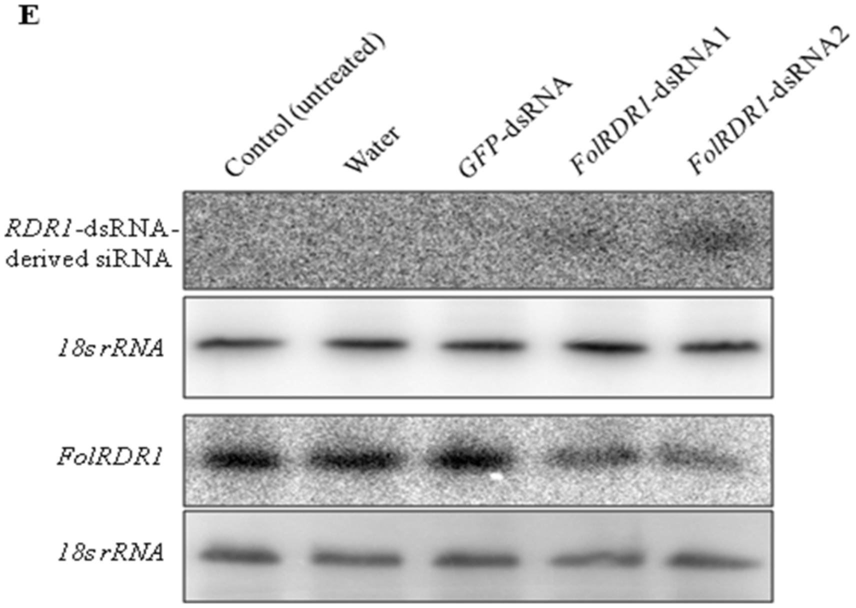

We also checked *FolRDR1*-dsRNA-derived siRNAs in *Fol*-infected tomato tissues. However, no signal was detected. We speculated that the proportion of *Fol* RNA in the total RNA extracted from plant was low due to the abundance of *Fol* biomass in tomato tissues. Therefore, no update was in Figure 6.

Part III – Minor Issues: Editorial and Data Presentation Modifications

Reviewer #1: (No Response)

Reviewer #2: (No Response)

Reviewer #3: The authors replied that for all the inoculation experiments, at least 30 tomato seedlings were inoculated for each treatment. However, it was “three biological replicates in

each experiment” in the revised figure legends of fig 2 and fig 6. Please be consistent in your description of the experimental data.

Response: Thanks for this comment, and we apologized for our confused state. We rephrased accordingly in the text.
